# The aerobic respiratory chain of *Pseudomonas aeruginosa* cultured in artificial urine media: Role of NQR and terminal oxidases

**DOI:** 10.1371/journal.pone.0231965

**Published:** 2020-04-23

**Authors:** Pingdong Liang, Xuan Fang, Yuyao Hu, Ming Yuan, Daniel A. Raba, Jie Ding, Dakota C. Bunn, Krithica Sanjana, Jun Yang, Monica Rosas-Lemus, Claudia C. Häse, Karina Tuz, Oscar Juárez

**Affiliations:** 1 Department of Biological Sciences, Illinois Institute of Technology, Chicago, IL, United States of America; 2 Carlson College of Veterinary Medicine, Oregon State University, Corvallis, OR, United States of America; National Research Council, ITALY

## Abstract

*Pseudomonas aeruginosa* is a Gram-negative γ-proteobacterium that forms part of the normal human microbiota and it is also an opportunistic pathogen, responsible for 30% of all nosocomial urinary tract infections. *P*. *aeruginosa* carries a highly branched respiratory chain that allows the colonization of many environments, such as the urinary tract, catheters and other medical devices. *P*. *aeruginosa* respiratory chain contains three different NADH dehydrogenases (complex I, NQR and NDH-2), whose physiologic roles have not been elucidated, and up to five terminal oxidases: three cytochrome *c* oxidases (COx), a cytochrome *bo*_3_ oxidase (CYO) and a cyanide-insensitive cytochrome *bd*-like oxidase (CIO). In this work, we studied the composition of the respiratory chain of *P*. *aeruginosa* cells cultured in Luria Broth (LB) and modified artificial urine media (mAUM), to understand the metabolic adaptations of this microorganism to the growth in urine. Our results show that the COx oxidases play major roles in mAUM, while *P*. *aeruginosa* relies on CYO when growing in LB medium. Moreover, our data demonstrate that the proton-pumping NQR complex is the main NADH dehydrogenase in both LB and mAUM. This enzyme is resistant to HQNO, an inhibitory molecule produced by *P*. *aeruginosa*, and may provide an advantage against the natural antibacterial agents produced by this organism. This work offers a clear picture of the composition of this pathogen’s aerobic respiratory chain and the main roles that NQR and terminal oxidases play in urine, which is essential to understand its physiology and could be used to develop new antibiotics against this notorious multidrug-resistant microorganism.

## Introduction

*Pseudomonas aeruginosa* is a Gram-negative, rod shaped γ-proteobacteria that colonizes a large diversity of environments, such as soil, water, and forms part of the normal microbiota of humans, animals and plants [1,2]. *P*. *aeruginosa* is also an opportunistic human pathogen that commonly infects epidermal burns [3], the lung epithelia of cystic fibrosis patients [4,5] and it is responsible for one third of all nosocomial urinary tract infections (UTI), which greatly increase the risk of morbidity and mortality in septic shock patients and diabetic patients [6] and are commonly associated with the use of contaminated catheters [7,8]. The World Health Organization ranks *P*. *aeruginosa* on its critical priority list as the second most important microorganism for the development of new treatments [9]. Thus, it is essential to understand the mechanisms used by this pathogen to survive in the urinary tract and other commonly infected environments.

**Fig 1:**
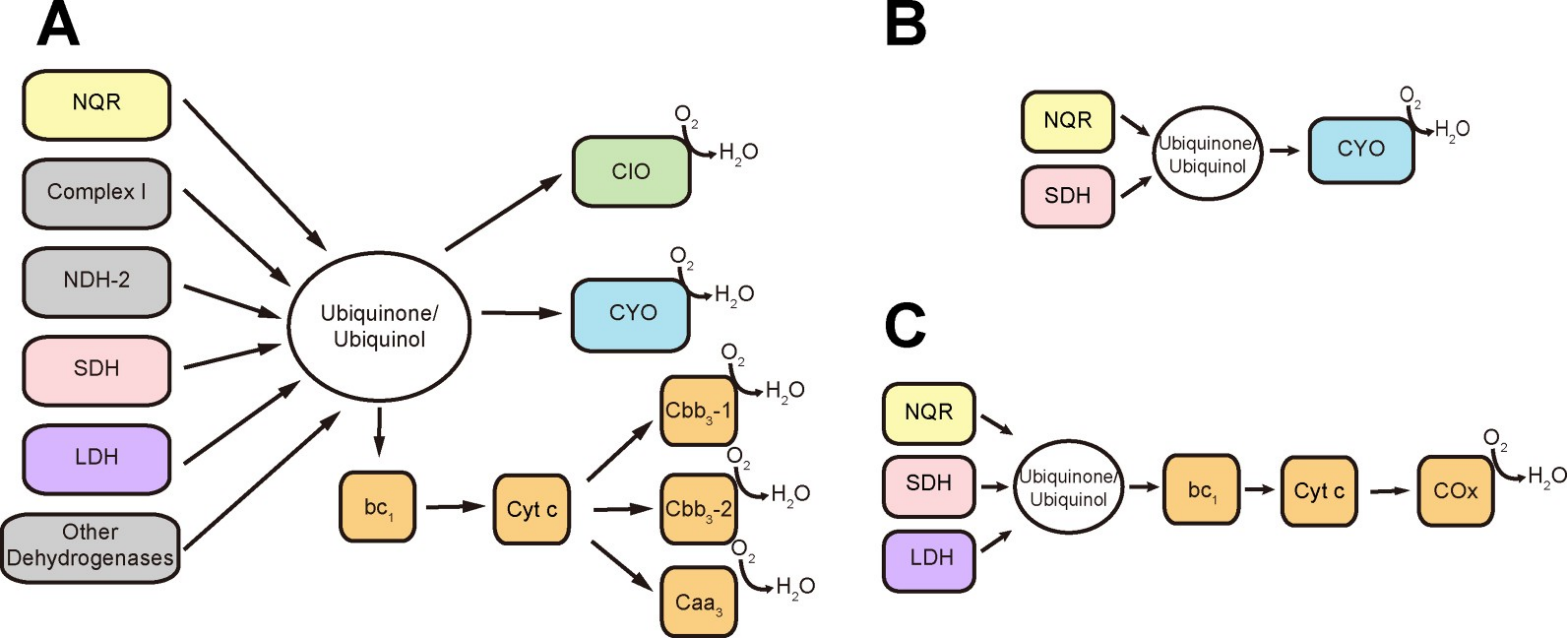
*P*. *aeruginosa* aerobic respiratory chain. A) Respiratory enzymes annotated in *P*. *aeruginosa* genome. Main components of the respiratory chain in LB (B) and mAUM (C) media.

*P*. *aeruginosa* has an enormous repertoire of molecular mechanisms that allow its adaptation to the environment, including a flexible metabolism that plays an essential role in its capability to colonize diverse niches. This bacterium is a facultative anaerobe with a highly branched respiratory chain that uses either oxygen or nitrogen oxides as final electron acceptors [10–12] and that under aerobic conditions uses oxidative phosphorylation, rather than fermentation, to produce ATP [4,11,12]. The genome contains 17 different dehydrogenases [13], 15 of which transfer the electrons from diverse reduced substrates to ubiquinone, and two of them transfer the electrons directly to nitrate reductases or cytochrome *c* [4,13]. The aerobic respiratory chain is also branched at the level of the ubiquinone pool, containing a cytochrome *bo*_3_ oxidase (CYO) and a cyanide-insensitive cytochrome *bd*–like oxidase (CIO), both of which transfer electrons directly from quinol to oxygen (Fig 1A) [12–14]. In addition to the quinol oxidases, the respiratory chain contains cytochrome *bc*_1_ and three cytochrome *c* oxidases (COx): *caa*_3_, *cbb*_3_-1 and *cbb*_3_-2 (Fig 1A) [4].

One of the most striking differences between the respiratory chain of this bacterium and that of human mitochondria is that *P*. *aeruginosa* possesses three different dehydrogenases that transfer electrons from NADH to the ubiquinone pool: complex I, NQR (formerly Na^+^-NQR, see below) and NDH-2 [13,15], while mitochondria only carry complex I. Complex I is a 500 kDa respiratory enzyme composed of 14 subunits and several cofactors: two 2Fe-2S centers, six 4Fe-4S clusters, and a non-covalently bound FMN [16,17]. NQR is composed of six subunits and contains one FAD, two covalently-bound FMNs, one 2Fe-2S center, a riboflavin molecule and possibly a non-heme Fe center [18–22]. NDH-2 is a 46 kDa peripheral protein with a single FAD cofactor [17,23]. The most important physiologic difference between these enzymes is that complex I is a proton pump, while NDH-2 is not involved in ion pumping [17,23]. Although in many cases it has been reported that NQR acts as a sodium pump, we recently described that *P*. *aeruginosa* NQR is an ion pump that specifically transports protons [24]. This raises questions regarding the role of each of these enzymes, especially since NQR and complex I appear to be functionally redundant, and NDH-2 does not participate directly in ion-gradient formation coupled to ATP synthesis. In other bacteria, the respiratory chain typically contains NDH-2 and either complex I or NQR [23,25–27], and the presence of all three NADH dehydrogenases is quite uncommon.

Although many studies have addressed the role of different respiratory complexes through mutagenesis, transcriptomics and genomics tools [13,28–32], a clear picture of how these complexes participate in the respiratory chain is currently unavailable. In this work, we performed systematic functional and kinetic characterizations of the aerobic respiratory chain of *P*. *aeruginosa* cultured in Luria Broth (LB) and modified artificial urine medium (mAUM), to understand the composition of the respiratory chain and its physiological role in cells growing in conditions that resemble urine. Our results indicate that *P*. *aeruginosa* respiratory chain composition is deeply influenced by the environmental conditions, which could be advantageous for its survival and could be important to establish a successful infection. For instance, while cytochrome CYO oxidase plays a major role in LB medium, the COx’s terminal oxidases play a dominant role in mAUM. Our results also show that NQR is the most important NADH dehydrogenase in both conditions. These studies are important to better understand the physiology of this important pathogenic bacterium and the role of the respiratory complexes in the survival in the urinary tract and in the initial steps of the colonization process, which may allow the identification of new therapeutic targets against *P*. *aeruginosa*.

## Materials and methods

### Modified artificial urine medium

The growth medium used in this study to simulate urine is a modification of the Artificial Urine Media reported by Brooks and Keevil [33]. The modifications are found in Table 1. One of the problems that we encountered with the original protocol is that precipitates were easily formed. Indeed, the authors indicate that precipitations were formed in AUM at temperatures higher than 25°C, which does not allow us to explore physiologic temperatures. Precipitation in mAUM was eliminated by adding 3 mM EDTA to the solution and by using MgCl_2_ instead of MgSO_4_. EDTA has shown antibacterial effects against *P*. *aeruginosa* planktonic cells and biofilms. However, these effects are blocked completely in the presence of calcium and iron [34]. As shown in Table 1, mAUM contains a molar excess of calcium, magnesium and iron compared to EDTA. Analysis of species concentrations using Chelator [35] indicates that the concentration of free EDTA is negligible in this medium, consistent with the healthy cell growth in these conditions. The concentration of phosphate was increased to 50 mM to serve as buffer, as the pH of the original medium spontaneously increased a few hours after preparation, probably due to the chemical decomposition of urea into ammonium. Finally, the concentration of uric acid was decreased to 0.23 mM, below its solubility limit (0.4 mM; 68 mg/ L) but within physiologic concentrations [36]. With these modifications we obtained a stable and reproducible medium that sustains *P*. *aeruginosa* growth and allows the study of this microorganism in conditions simulating human urine.

**Table 1:** Comparison of artificial urine and modified artificial urine media.

	[Nutrient]	
	AUM	mAUM
**EDTA**	-	3 mM
**Citric acid**	2 mM	2 mM
CaCl_2_	2.5 mM	2.5 mM
**NaCl**	90 mM	80 mM
Na_2_SO_4_	10 mM	10 mM
MgSO_4_	2 mM	-
MgCl_2_	-	5 mM
KH_2_PO_4_	7 mM	50 mM
K_2_HPO_4_	7 mM	-
**Uric acid**	0.4 mM	0.23 mM
NaHCO_3_	25 mM	37 mM
**Peptone L37**	1 (g/l)	-
**Bovine peptone**	-	1 (g/l)
**Yeast extract**	0.5 (g/L)	0.5 (g/L)
**Urea**	170 mM	170 mM
**Creatinine**	7 mM	7 mM
NH_4_Cl	25 mM	25 mM
**Lactic acid/ Sodium lactate**	1 mM	1 mM
FeSO_4_	5 μM	3.5 μM
**pH**	6.5	6.5

### *P*. *aeruginosa* growth

Overnight cultures of *P*. *aeruginosa* PAO1 grown in LB broth media were washed with saline solution (0.9% NaCl). The washed cells were inoculated (1:1000 dilution) into LB broth or mAUM and were grown under agitation (250 rpm) at 37°C. Bacterial growth was monitored by measuring OD_595_. The growth curves were fitted to a logistic function (Eq 1) [37] to calculate the growth rate (μ), maximum growth (*a*) and lag phase (*l*).

$$y=\frac{a}{1+e^{\frac{4\mu }{a}\left(l-t\right)+2}}$$ (1)

### *P*. *aeruginosa* membrane preparation

*P*. *aeruginosa* PAO1 cells were harvested at the early stationary phase of growth, washed twice with KHE buffer (150 mM KCl, 20 mM HEPES, 1 mM EDTA, pH 7.5) and stored at -80°C. Frozen cell pellets were thawed, resuspended in KHE buffer supplemented with 10 μg/ ml DNAase I, 5 mM MgCl_2_ and 1 mM PMSF, and passed twice through an Emulsiflex-C5 high pressure homogenizer (Avestin) at 16,000 psi. Cell debris was eliminated by centrifugation at 10,000 × *g* for 30 min at 4°C. The supernatant was collected and ultracentrifuged at 100,000 × *g* for 5 h. The pellet, containing the membranes, was washed and resuspended in KHE buffer and stored at -80°C.

### *P*. *aeruginosa* NQR purification

*P*. *aeruginosa* NQR was purified as described previously [24]. Briefly, *P*. *aeruginosa nqrA-F* NQR operon was cloned in pBAD plasmid and transformed into *Vibrio cholerae* O395N1 *Δnqr* cells [38]. The protein complex was expressed (upon addition of arabinose to the culture media) and was purified from cytoplasmic membranes after solubilization with β-D-dodecylmaltoside (DDM) and Ni-NTA affinity chromatography, followed by anion exchange chromatography, using DEAE-Sepharose [38]. Protein concentration for these and other samples was measured by the BCA assay. NQR concentration was measured spectrophotometrically at 450 nm in a Gd-Cl denatured sample, as described before [38].

### Blue native gel electrophoresis and NADH dehydrogenases in gel activity

Proteins were separated by blue native gel electrophoresis, as described by Schagger *et al*. [39]. Briefly, the membranes were solubilized in buffer containing 750 mM ε-aminocaproic acid, 50 mM Bis-Tris, pH 7 and DDM (2 g/g prot) for 30 min on ice. The suspension was centrifuged at 100,000 x *g* for 40 min and the supernatant, containing the solubilized membrane proteins, was collected, mixed with 3x running buffer (with Coomassie blue G-250) and loaded in the gel’s well. The samples were resolved in 4–16% polyacrylamide gradient gel. In-gel NADH dehydrogenase activity staining was performed as reported previously [40]. BN-PAGE gel lanes were sliced and incubated at room temperature in buffer containing 100 mM Tris-HCl, 140 μM NADH, 50 μM MTT, pH 7.4. After activity staining, the excess of background Coomassie blue was removed by washing the gel in 0.1% SDS.

### Second dimension SDS- PAGE

BN-PAGE bands containing NADH dehydrogenase activity were excised and incubated for 30 min in 60 mM Tris-HCl, pH 6.8, 1% SDS, 1% β-mercaptoethanol. The gel pieces were washed twice with 25 mM Tris, 192 mM glycine, 0.1% SDS and then mounted on a 15%-SDS-PAGE. The gel was exposed to UV light to identify the fluorescent bands corresponding to NQR subunits C and B containing the covalently-bound FMN [41]. The gel was then stained with Coomassie blue.

### Oximetry

The respiratory activity of *P*. *aeruginosa* membranes (0.2 mg of protein /mL) was measured at 37°C in KHE buffer in a 2 mL custom-made glass chamber, adapted to a Clark-type oxygen electrode (YSI 5300), as described previously [42]. Assays were carried out in the presence of specific substrates and inhibitors for each of the respiratory complexes. NADH oxidase activity was tested using 200 μM NADH. Succinate dehydrogenase was tested in presence of 20 mM succinate. The activity of other dehydrogenases was measured with 10 mM D, L- malate, 10 mM L-lactate, 10 mM glucose or 0.3% ethanol. Quinol oxidase activity was assessed with 50 μM ubiquinone-1 (2,3-Dimethoxy-5-methyl-6-(3-methyl-2-butenyl)-1,4-benzoquinone, Sigma-Aldrich) or menaquinone-2 (2-(3,7-dimethyl-2,6-octadienyl)-3-methyl-1,4-Naphthoquinone, Sigma-Aldrich) in the presence of 500 μM DTT, which readily reduces these quinones [43–46]. The activity of cytochrome *c* oxidases was tested with the artificial redox couple 100 μM TMPD^+^ and 5 mM ascorbic acid.

### KCN titration analysis

The NADH-dependent respiratory activity of the two types of membranes was measured in the presence of different concentrations of KCN, freshly prepared in KHE buffer. Initial activity rates were plotted against KCN concentrations and the data was fitted to the equation of three independent enzymes competitively inhibited by KCN. The equation for each of these enzymes has the familiar form: *v = Vmax* ([S]/*Km*)/ (1 + ([S]/ *Km*) + ([I]/ *K*_*iapp*_), where *Vmax* is the maximum activity of the enzyme, *K*_*iapp*_ is the apparent inhibition constant and [S]/ *Km* is the substrate’s saturation ratio (assumed to be close to saturating, >10).

### NADH dehydrogenase activity

*P*. *aeruginosa* membranes were solubilized with 0.3% DDM for 30 min at 4°C and the suspension was ultracentifuged at 100,000 x *g* for 1 h. The supernatant was used to measure the NADH-dependent ubiquinone reduction at 282 nm [38] in KHE buffer containing 50 μM ubiquinone-1 and 250 μM NADH or 250 μM deamino NADH.

## Results

### *P*. *aeruginosa* growth in LB and mAUM

*P*. *aeruginosa* PAO1 strain was cultured aerobically in modified artificial urine media (mAUM) (see Materials and Methods), to simulate the conditions that the planktonic state of this microorganism might encounter in the urinary tract or in catheters when they start the colonization process. Previous studies showed that the urinary tract environment is aerobic, with urine’s oxygen concentration ranging from 130 to 90 μM in healthy patients *vs* patients with urinary tract infections [47]. Moreover, the microorganisms associated with these infections are aerobic or microaerophilic [48]. However, some studies indicate that in certain conditions urinary tract infections could be microaerophilic [49,50].

**Fig 2:**
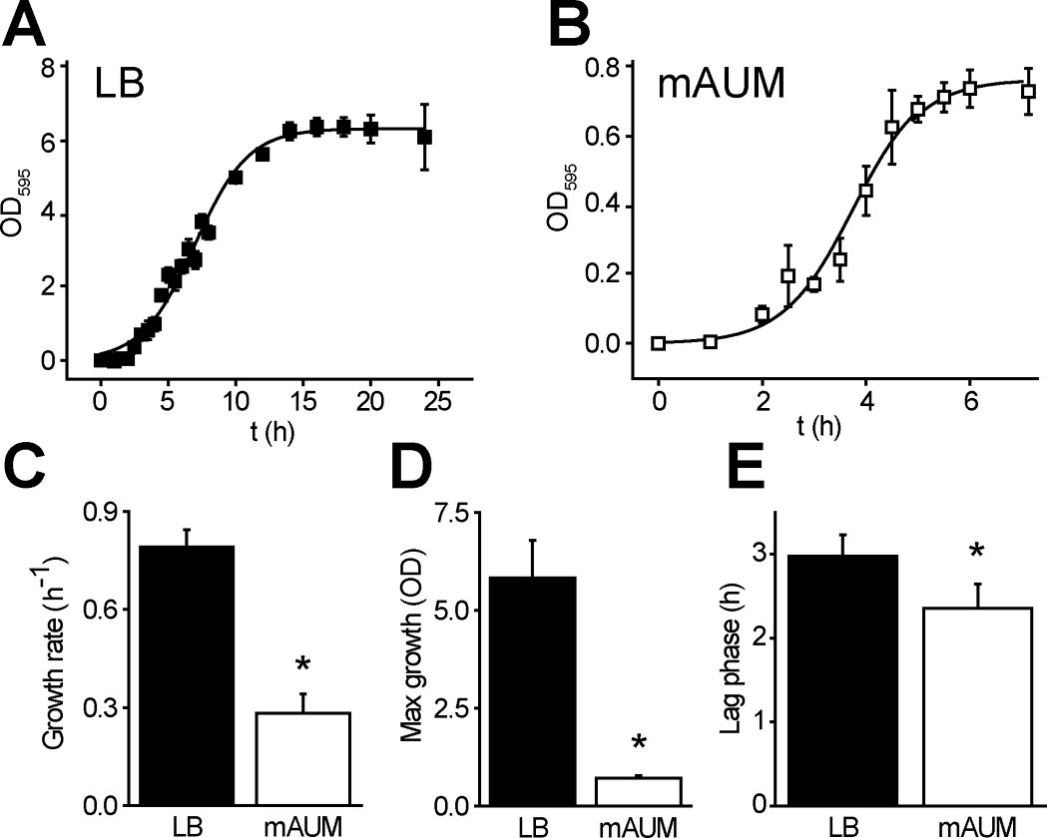
*P*. *aeruginosa* growth in LB and mAUM. *P*. *aeruginosa* growth curves in LB (A) and mAUM (B) media. Comparison of growth parameters between LB and mAUM: growth rate (C), maximum growth (D) and lag phase (E). These parameters were calculated using Eq 1. Results are expressed as mean ± S.D. n ≥ 5. *, p<0.01 after t test.

mAUM medium is based on the previously published AUM [33] with some modifications to improve the solubility of salts, reduce precipitations and drastic changes in pH (Table 1, see Materials and Methods). The bacterial growth obtained in mAUM (Fig 2B) was compared to the growth in standard laboratory Luria Broth medium (Fig 2A). *P*. *aeruginosa* growth curves were analyzed using a logistic function (Eq 1, Materials and Methods) [37]. The main parameters obtained from this analysis are the duplication rate (μ) (Fig 2C), total growth (a) (Fig 2D) and length of the lag phase (l) (Fig 2E). Although significant differences were found in all three parameters, the major changes were found in the growth rate and total growth, which were 3 and 7 times higher in LB media *vs* mAUM, respectively. Due to the changes in growth kinetics, the stationary phase was reached at 15 h in LB (Fig 2A) and at 6 h in mAUM (Fig 2B). The growth rates in LB reported here are similar to the growth rates reported in other works [31]. Importantly, these data indicate that *P*. *aeruginosa* actively grows in these conditions and does not enter a dormant state or immediately produces biofilms when exposed to urine.

### Organization of *P*. *aeruginosa* respiratory chain in mAUM *vs* LB media: Dehydrogenases and quinones

**Fig 3:**
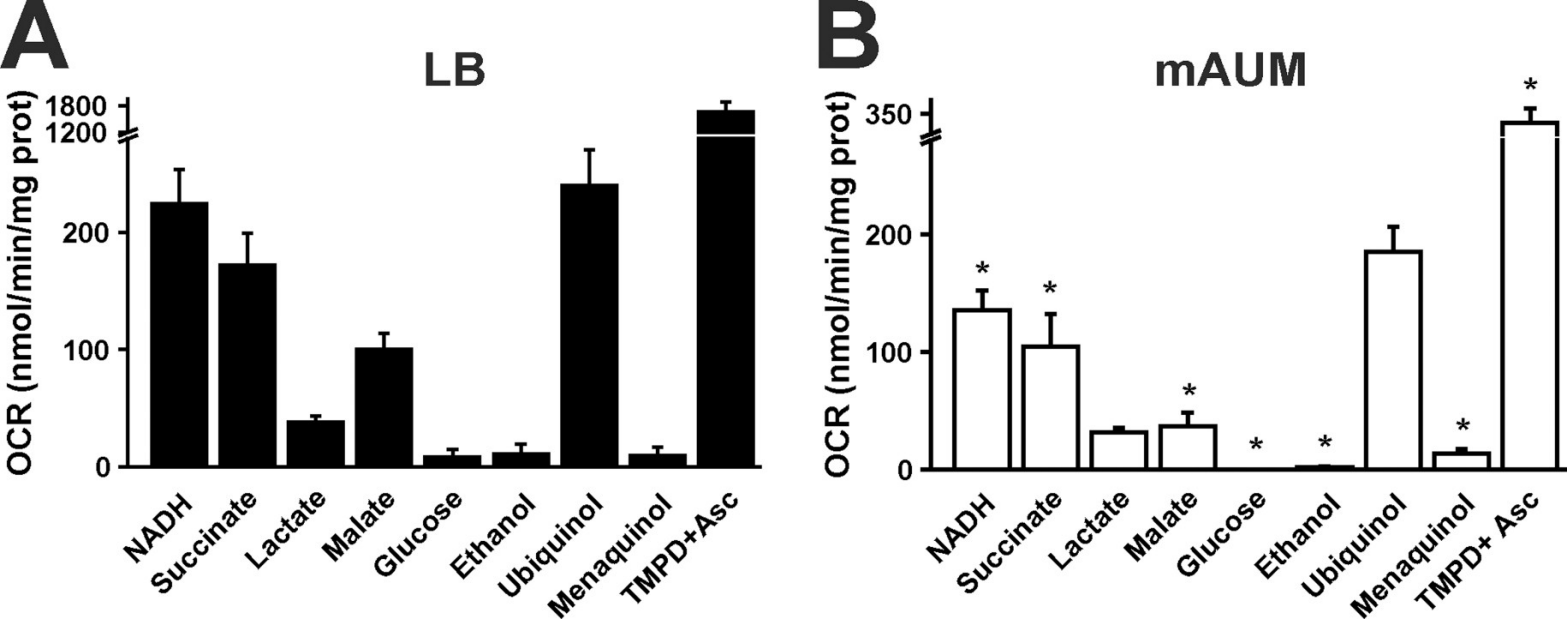
*P*. *aeruginosa* respiratory activity in LB and mAUM membranes. Oxygen consumption rate (OCR) of membranes obtained from bacteria grown in LB (A) or mAUM (B). Oxygen consumption was measured in KHE buffer in the presence of the next substrates: 200 μM NADH, 10 mM succinate, 10 mM lactate, 10 mM glucose, 0.3% ethanol, 50 μM ubiquinol or 50 μM menaquinol or 100 μM TMPD^+^ plus 5 mM ascorbic Acid. Bars represent mean ± S.D. n ≥ 3. *, p<0.05 after t test between LB and mAUM.

To understand the metabolic adaptations of *P*. *aeruginosa* in conditions that mimic human urine, cells were cultured in mAUM and LB media and harvested at the early stationary phase of growth. To characterize the aerobic respiratory chain, oxygen consumption rates (OCR) were measured in purified plasma membranes. OCR activities were measured in the presence of different substrates: NADH, succinate, lactate, malate, glucose and ethanol. Moreover, experiments were carried out with ubiquinol or menaquinol to identify the type of quinone used by the cells. Fig 3A and 3B shows the dehydrogenase activities of *P*. *aeruginosa* membranes in each growth media. As can be observed, NADH dehydrogenase and succinate dehydrogenase activities are 40–50% smaller in mAUM (Fig 3B) *vs* LB (Fig 3A). Malate dehydrogenase also show differences, with higher activities in LB medium (Fig 3A and 3B). On the other hand, lactate dehydrogenase activity was not modified in these conditions. Glucose oxidase and ethanol dehydrogenases were also tested but have minor activities compared to the other dehydrogenases.

In order to determine the type of quinone used as electron carrier, we tested the respiratory activity in presence of ubiquinol-1 and menaquinol-2. Fig 3 shows that the ubiquinol oxidase activity is similar compared to the NADH dehydrogenase activity in LB (Fig 3A) and mAUM (Fig 3B), while menaquinol oxidase activity is negligible in both types of media. The data indicate that ubiquinone is the preferred electron carrier in the respiratory chain of this bacterium.

### Composition of *P*. *aeruginosa* respiratory chain in mAUM *vs* LB media: Terminal oxidases

As mentioned above, in addition to cytochrome *bc*_1_ and three cytochrome *c* oxidases (COx), *P*. *aeruginosa* contains two different ubiquinol oxidases: CIO and CYO [12,28,30]. To understand the role of these complexes in these growth conditions, the respiratory activity obtained with ubiquinol was compared to the rate of oxygen consumption in presence of ascorbic acid and TMPD^+^. Ascorbate-TMPD^+^ chemically reduce the endogenous cytochrome *c* and is used as substrate by COx oxidases [51]. TMPD-ascorbate oxidase activity is 6 and 2 times higher compared to the ubiquinol oxidase activity in LB (Fig 3A) and mAUM (Fig 3B) membranes, respectively. These results could suggest that in both media COx oxidases play a major role. However, these measurements cannot be used to predict the activity *in situ*, since COx activity was assayed by fully-reducing cytochrome c (representing near-maximal activities) and reduced cytochrome c concentration could be far from optimal under physiologic conditions.

**Fig 4:**
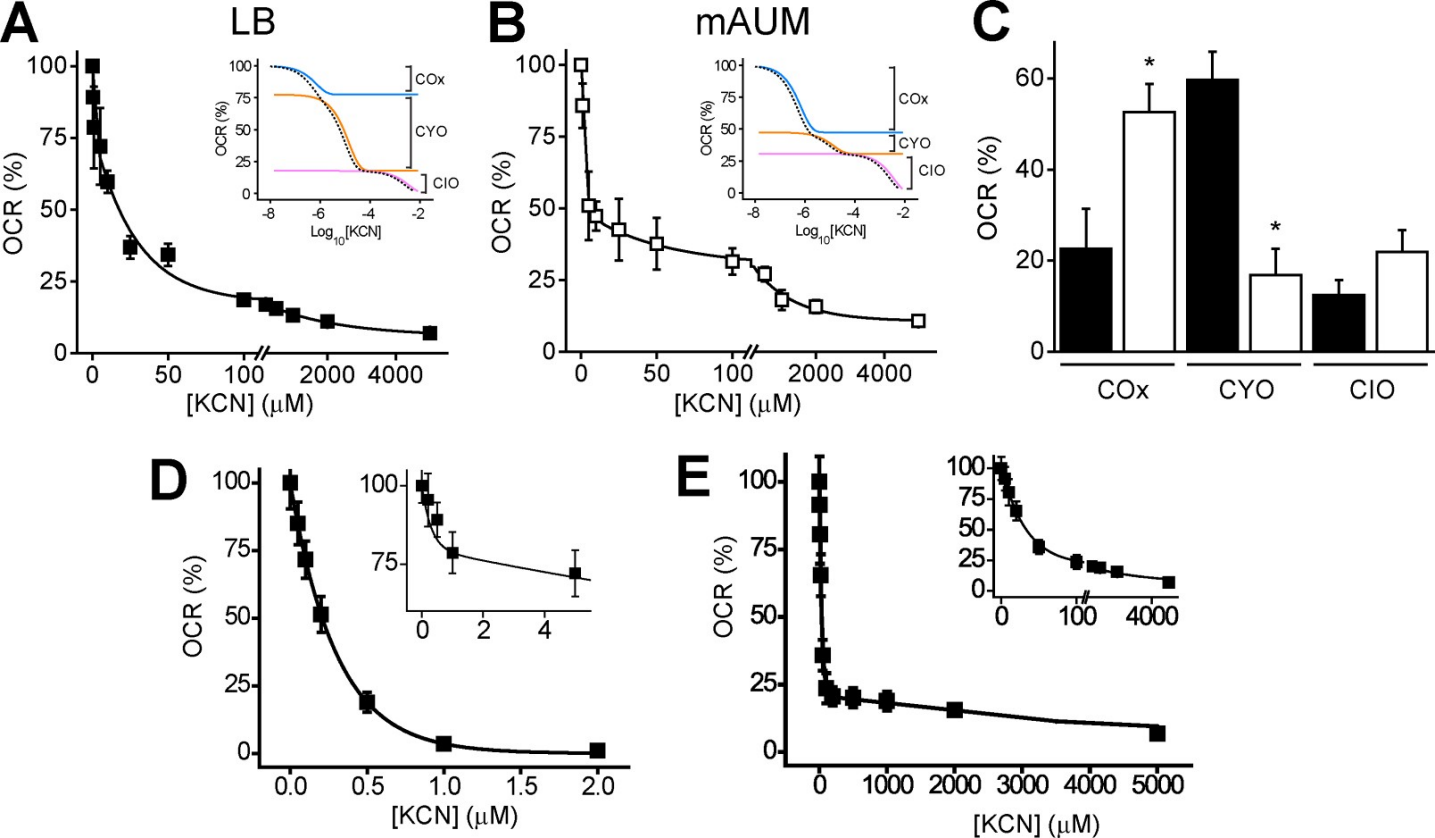
Cyanide titration of respiratory activity. NADH-dependent respiratory activities were measured in LB (A) and mAUM (B) membranes in the presence of different concentrations of KCN (0–5 mM). Titration curves were fitted to the function with three independent Competitive Inhibition Michaelis-Menten components (see Materials and Methods). The first component (*K*_*iapp*_<1 μM) was assigned as COx, the second component (*K*_*iapp*_ 10 μM) was assigned as CYO and the third component (*K*_*iapp*_ 2.5 mM) was assigned as CIO. The activities (*Vmax*) of these components in LB (black bar) and mAUM (white bar) are compared in panel C. Bars represent mean ± S.D. n ≥ 3. *, p<0.01 after t test. Insets in panels A and B show the expected titration curves of COx (blue), CYO (orange) and CIO (pink) oxidases, and the addition of the three components (dashed black line). The colored lines were slightly moved to the right for clarity. D) Titration of the Ascorbate /TMPD respiratory activity of LB membranes. The fitting line was obtained with a single-component kinetic model. The inset shows a magnification into the 0–5 μM KCN region of panel A. E) KCN titration of the ubiquinol-dependent respiratory activity in the presence of 1 μM antimycin A, which inhibits cytochrome *bc*_1_. The fitting line was obtained with a two-component kinetic model, with *K*_*iapp*_ values of 15 μM and 3 mM. The inset shows a replotting of the data with a break from 100–200 μM KCN.

To measure the relative contribution of the three types of oxidases to cell physiology, the respiratory activity was measured in the presence of different concentrations of KCN. The terminal oxidases have very different cyanide sensitivities, which can be used as a tool to quantify their relative contributions to respiration. As shown in Fig 4, the KCN titration of the NADH-dependent respiratory activity revealed three distinct kinetic components for LB (Fig 4A) and mAUM (Fig 4B) membranes, with apparent inhibition constants (*Ki*_*app*_) that were at least one order of magnitude higher, producing step ladder-like curves. The first component is highly sensitive to cyanide, with a *Ki*_*app*_ of 0.5–1 μM, consistent with the high affinity of COx oxidases for this inhibitor [52,53], including those of *Pseudomonas* species [54]. The second component has a *Ki*_*app*_ of 10 μM, similar to the reported values for CYO oxidase (8 μM) [55,56]. The last component has a *Ki*_*app*_ of 2.5 mM, and it is most likely due to CIO oxidase activity, which is highly resistant to cyanide [32,56]. To corroborate that *P*. *aeruginosa* terminal oxidases have similar sensitivities for cyanide as other members of these families, we carried out a KCN titration of the COx and ubiquinol oxidases in LB membranes (Fig 4D and 4E). COx activity was measured using Ascorbate-TMPD, as described previously. Fig 4D shows that the COx activity is highly sensitive to KCN, with a *K*_*iapp*_ of 0.3±.1 μM, which corroborates that the first kinetic component in Fig 4A (magnified in the Fig 4D inset) is indeed COx. Ubiquinol oxidase activity was measured with ubiquinol- DTT in the presence of 1μM antimycin A, which specifically inhibits complex *bc*_1_ and indirectly inhibits COx. The titration of the activity revealed two components, with *K*_*iapp*_‘s of 15 μM and 3 mM, likely corresponding to CYO and CIO.

The triphasic behavior found in Fig 4A and 4B (with similar *K*_*iapp*_’s as found here) has been reported in other species, such as *Pseudomonas pseudoalcaligenes* [54] and it is also evident in the re-plotting of the original data on *P*. *aeruginosa* respiration by Cunningham, *et al*. [32]. In previous reports cyanide titration data was analyzed using the empirical equation: A/ 1+([I]/ IC_50_)^n^ [56], which it is not based on a strict kinetic model and incorporates a “Hill coefficient” (n), providing a seemingly good data fitting but no strict biochemical meaning. In this report, the titration curves were analyzed with an equation that considers three independent kinetic Michaelis-Menten components, with individual *Ki*_*app*_ and *Vmax* values (see Materials and Methods), showing excellent fitting to the data. Insets in Fig 4A and 4B show the predicted activities of each kinetic component when varying the cyanide concentration, to better illustrate the analysis carried out. As can be observed, the first (COx) and the second (CYO) components are saturated by cyanide concentrations of 10 and 100 μM, while and the third component (CIO) is active at concentrations in the mM range. Fig 4C shows the comparison of the activities (*Vmax*) of the three components, as can be observed the three types of oxidases have significant contributions to the respiratory activity in both cases. However, the relative contribution of each oxidase varies depending on the culture medium. In LB medium 50% of the activity can be attributed to CYO oxidase, while in mAUM more than 60% of the activity can be attributed to COx. This difference could be related to the importance of energy balance in the relatively poorer urine medium (see below).

### Composition of *P*. *aeruginosa* respiratory chain in mAUM *vs* LB media: Role of NADH dehydrogenases

**Fig 5:**
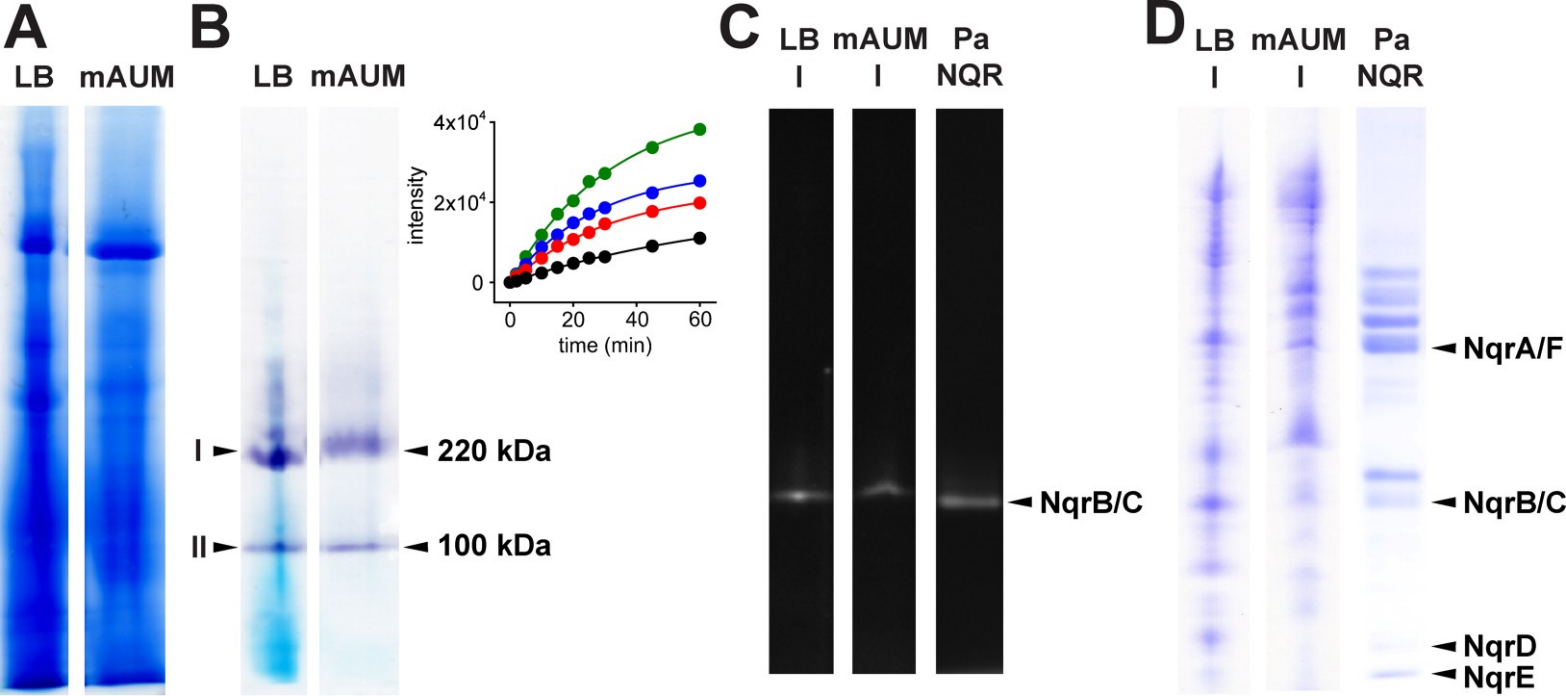
BN and 2D-SDS PAGE identification of NQR in LB and mAUM membranes. A) Coomassie-stained BN-PAGE gel of lanes containing solubilized LB and mAUM membranes. B) In-gel NADH dehydrogenase activity of BN-PAGE lanes of LB and AUM membranes, showing the 100 and 220 kDa bands. Inset, densitometry assay of the in-gel kinetics of NADH dehydrogenase activity (Black, LB -100 kDa; Red, AUM-100 kDa; Blue, LB-220 kDa; Green, AUM-220 kDa). The 220 kDa bands of the BN-PAGE gel were excised and run in a second dimension SDS-PAGE. The 2D gel lanes were exposed to UV light to identify the fluorescent bands of NQR subunits B and C (panel C), which co-migrate in this type of gel, due to the high hydrophobicity of subunit B, and were subsequently Coomassie-stained (D). Purified Pa-NQR [24] was run as standard for comparison.

Three different NADH dehydrogenases are annotated in the genome of *P*. *aeruginosa*: complex I, NDH-2 and NQR. The presence of the three types of NADH dehydrogenases is puzzling, especially since NQR and complex I are both proton pumps [24,57] and NDH-2 is not linked to the generation of an electrochemical gradient. Here we aim to elucidate the roles of these enzymes in the physiology of *P*. *aeruginosa*. Although these enzymes catalyze the same redox reaction, they can be distinguished by their molecular weights, using blue native gel electrophoresis (BN-PAGE), and by their inhibitor sensitivity and substrate specificity. Membranes of cells grown in LB and mAUM were solubilized using n-dodecyl-β-D-maltoside (DDM) (2 g /g prot). The proteins were separated using BN-PAGE and the NADH dehydrogenase activity was assayed in-gel, using NADH as substrate and MTT (3-(4,5-dimethylthiazol-2-yl)-2,5-diphenyltetrazolium bromide) as an artificial electron acceptor. The reduction of MTT produces a formazan precipitate and purple bands appear in the gel in locations where the NADH dehydrogenase reaction takes place. Two NADH dehydrogenase bands of 100 and 220 kDa (Fig 5B) were observed in both types of membranes, matching the molecular weights of a NDH-2 dimer (46 kDa monomer) and the six-subunit NQR complex (210 kDa) [24]. To estimate the relative activity of both bands, the reaction was measured densitometrically at different time points (Inset Fig 5B). From the initial rates we determined that the 220 kDa band is 3–5 times more active than the 100 kDa band. A third band, with an estimated molecular weight of 500 kDa (likely corresponding to complex I [16,23]), could also be observed in LB membranes, but the incubation time required to visualize this band was much longer (overnight) compared to the other bands. The 220 kDa BN-PAGE bands were excised and ran in a second dimension SDS-PAGE. The gel was exposed to UV light a fluorescent bands were observed in the lanes of the 220 kDa complex, corresponding to the covalently-attached FMN to subunits B and C of NQR (Fig 5C) [41,58], as confirmed by mass spectrometry analysis (Proteomics Unit, University of Illinois at Chicago) of the fluorescent band (S1 Table). The 220 kDa band also contain the six subunits of the NQR complex, as shown in the Coommassie-stained 2D SDS-PAGE gel (Fig 5D), confirming the presence of this enzyme in both conditions.

The 2D-SDS PAGE of the 100 kDa band contained a band with the expected molecular weight of NDH-2 of 46 kDa, which was excised and analyzed by mass spectrometry. Several proteins were identified but none of them corresponded to NDH-2 (S1 Table). Although the 100 kDa band has significant activity in gel, it is not related to any enzyme of the respiratory chain. It should be pointed out that the turnover rates of NQR (500 s^-1^ [38]) and NDH-2 (900 s^-1^ [59]) are comparable and based on NQR abundance in the gel, NDH-2 should be an abundant band as well, if the 100 kDa activity band contains this protein.

**Fig 6:**
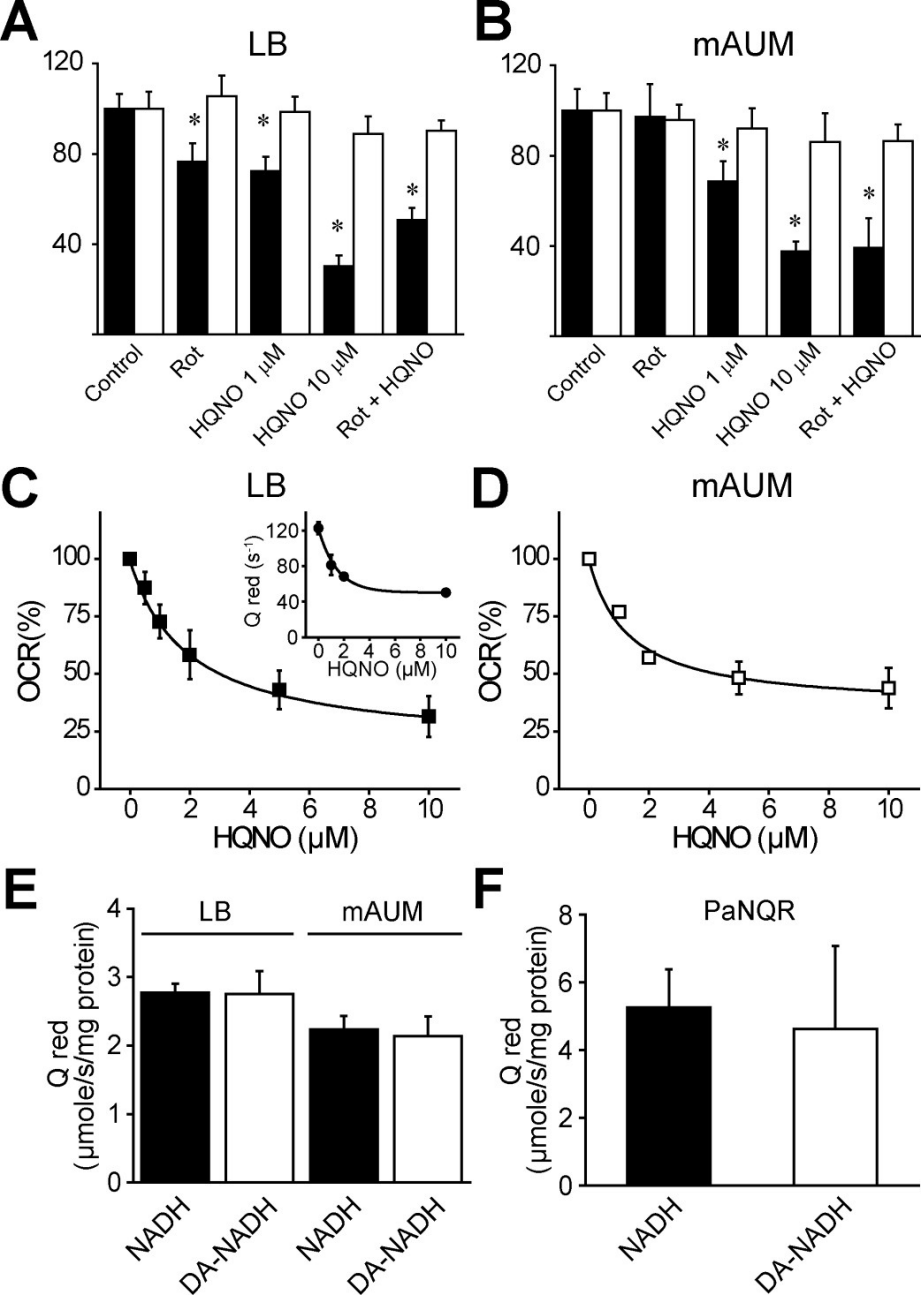
NQR activity measurements in LB and mAUM membranes. NADH-dependent (black) and Succinate-dependent respiratory activity of LB (A) or mAUM (B) membranes in the presence of rotenone (1 μM) or HQNO (1 or 10 μM). Bars represent mean ± S.D. n ≥ 3. *, p<0.05 after t test between NADH-dependent (black) or Succinate-dependent (white) respiratory activity. Effect of HQNO on the NADH-dependent rate of oxygen consumption in LB (C) and mAUM membranes (B). Inset in C, HQNO titration on the activity of the purified Pa-NQR. Inset data were taken from reference [24]. Ubiquinone reductase activity of solubilized membranes obtained from cells grown in LB or mAUM (E) or purified *P*. *aeruginosa* NQR (F) using as substrates NADH (black) or deamino NADH (white; DA-NADH). Data are expressed as SDM (n ≥ 5). * p < 0.05 vs control.

Although in-gel activity measurements indicate that NQR is the main NADH dehydrogenase, the effects of specific inhibitors of complex I (rotenone) [60] and NQR (HQNO, N-2-heptyl-4-hydroxyquinoline) [61,62] were tested on the rate of oxygen consumption in membranes obtained from LB or mAUM media to confirm this hypothesis. At a saturating concentration, rotenone (1 μM) (*K*_i_ = 4 nM) [63,64] inhibits <20% of the respiratory activity of LB membranes (Fig 6A), and had no effects on the activity of mAUM membranes (Fig 6B), indicating that complex I does not participate in the respiratory activity in mAUM, consistent with the low activity obtained in in-gel assays. The titration of the NADH-dependent activity with HQNO produced a curve with a *K*_i_ = 2 μM and a kinetic component of 40% that is insensitive to concentrations >10 μM of this inhibitor, in both types of membranes (Fig 6C and 6D). This behavior is nearly identical to the titration of the purified *P*. *aeruginosa* NQR (Fig 6C inset) [24]. To test that HQNO and rotenone act specifically on the NADH dehydrogenases, these inhibitors were tested using succinate as a substrate, as it has been reported that HQNO can inhibit other respiratory enzymes [65–67]. As shown in Fig 6A and 6B, the inhibitors had little effect on the activity with succinate in both types of membranes, indicating that at these concentrations they act specifically on the NADH dehydrogenases, in particular on NQR.

Finally, to corroborate that the NADH-dependent respiratory activity in membranes is mostly due to NQR and not to NDH-2, we tested the ubiquinone oxidoreductase activity in membranes solubilized with DDM using NADH or diamino-NADH as substrates. While NQR can use both substrates equally well (Fig 6F) [68,69], NDH-2 can only use NADH [70,71]. The ratio of activity with NADH and deamino-NADH can be used to determine the contributions of these two enzymes. As shown in Fig 6E, the activities obtained with these two substrates are nearly identical in both types of membranes. Taken together the data unambiguously demonstrate that NQR is the main NADH dehydrogenase for *P*. *aeruginosa* in the conditions tested in this study.

## Discussion

Previous studies using proteomics, transcriptomic and mutagenesis methodologies have addressed the role of different respiratory complexes for this microorganism [13,28–32]. However, these methodologies have intrinsic limitations and a clear understanding of the specific roles of respiratory enzymes used by *P*. *aeruginosa* in diverse conditions is missing. Among the aspects that limit the usefulness of these techniques are: 1) transcriptomics studies normally report the relative expression of a gene compared with a specific “basal” condition and mRNA content does not necessarily directly correspond to protein content, 2) proteomic identification and quantification may not be used to estimate the enzymatic activity in the pathway, due in part to the presence of inactive and active pools of enzymes (apo vs holo) and because protein content does not necessarily linearly correlate with enzymatic activity, due to multifactorial regulation. On the other hand, mutagenesis studies are a powerful tool to analyze the effects of gene elimination on microbial growth parameters. However, this technique relies on the ability of cells to grow in the absence of specific genes, and it is highly likely that this method could select variants that can withstand genetic manipulation by upregulating other genes or pathways, which might not represent the wild-type genetic background. In this report we performed a comprehensive functional characterization of the respiratory chain of *P*. *aeruginosa*, measuring directly the metabolic and enzymatic activities within their pathways. Although the method is disruptive to the cell, it allows the direct determination of the activities of different respiratory complexes using their specific kinetic properties, including substrate and inhibitor specificity. Our results allow us to reconstruct the electron transfer pathway used by *P*. *aeruginosa* planktonic state while growing in medium similar to human urine.

### Role of succinate dehydrogenase, malate dehydrogenase and lactate dehydrogenase in carbon flux

Previous studies have shown that the Krebs and glyoxylate cycles are highly active in *P*. *aeruginosa* isolated from both cystic fibrosis patients and from urinary tract infections [72]. Here we have found that succinate dehydrogenase is very active in both LB and mAUM membranes, which is consistent with a high carbon flux through these pathways, allowing the synthesis of important molecules for bacterial survival [72]. In addition, we report an important respiratory activity with malate in both LB and mAUM membranes, which indicates that the membrane-bound malate dehydrogenase [73] found in the genome is active. *P*. *aeruginosa* genome also contains two genes for membrane-bound lactate dehydrogenases (mLDH), which transfer the electrons to ubiquinone, rather than to NAD as the soluble enzyme does [74]. The relatively high activity of mLDH found in mAUM membranes could be advantageous for the growth in urine medium, which contains high amounts of the racemic mixture of lactate, the products of human (L-lactate) and microbial metabolism (D-lactate) [75].

### Role of terminal oxidases in artificial urine medium

Although, P. aeruginosa is an important human pathogen that produces one of the most common hospital-acquired infection, the regulation and physiologic roles of *P*. *aeruginosa* terminal oxidases during urinary tract infections have remained completely unknown. Previous reports suggested that CYO is not a critical enzyme for the aerobic metabolism of *P*. *aeruginosa*, since its expression is low in LB medium and it seems to be induced specifically during iron starvation [76]. Moreover, mutants that lack this enzyme do not show major changes in the growth parameters [28]. However, our results unambiguously indicate that CYO is the main terminal oxidase in LB medium, contributing to 50% of the respiratory activity. These findings highlight the need to complement mutagenesis, proteomic and transcriptomic data with functional analysis of the enzymes and metabolic pathways. Interestingly, in other gram-negative proteobacteria, such as the closely-related *Pseudomonas putida* [77], and also in *Escherichia coli* [78] as well as in *Gluconobacter oxydans* [79], CYO was also found to be the main terminal oxidase, suggesting a common evolutionary or physiologic trend among prokaryotes.

On the other hand, in mAUM membranes 60% of the activity can be attributed to COx oxidases, since most of the activity is sensitive to very low cyanide concentrations (< 1μM). Among the three different types of terminal oxidases, COx oxidases (c*aa*_3_, *cbb*_3_-1 and *cbb*_3_-2) provide the highest energy yields to the cell. COx oxidases could be favored in mAUM because it is not a rich medium compared to LB and in these conditions cell energetics could have a predominant role. Cytochrome *cbb*_3_-1 oxidase may be the main terminal oxidase used in these conditions, since it is constitutively expressed and appears to be a major component during exponential growth. On the other hand, *cbb*_3_-2 oxidase appears to be induced in the stationary phase when the oxygen tension drops [80] and previous studies have shown that c*aa*_3_ oxidase expression is extremely low under aerobic conditions [76]. Recent studies have shown that *cbb*_3_ oxidases are important for the formation of biofilms by *P*. *aeruginosa*, which strongly suggests that this terminal oxidase plays major roles during the colonization of lung epithelium [81] and as shown in this work, also in the colonization of the urinary tract or catheters. As shown in Fig 4, both types of membranes have a significant cyanide-resistant respiratory activity, which can be attributed to CIO oxidase [12,82–84]. CIO oxidase expression has been considered a pathogenicity marker for *P*. *aeruginosa* [82], since this microorganism actively produces cyanide during infection [28], which can be as high as 300 μM in culture [32].

### Role of NQR in *P*. *aeruginosa* physiology: Autopoisoning resistance

Recent studies show an increased expression of complex I and NQR in *P*. *aeruginosa* isolated from cystic fibrosis patients [85]. Moreover, mutagenesis analyses indicate that complex I is critical for the survival of *P*. *aeruginosa* under microaerophilic conditions [15,86]. However, the role of NADH dehydrogenases in the physiology and pathogenic behavior of this bacterium remains almost completely unknown. In this work we have determined that *P*. *aeruginosa* NADH dehydrogenase is highly active in LB and mAUM. In both media most of the NADH dehydrogenase activity is resistant to rotenone, indicating that complex I does not play a major role in the respiratory activity. To test if NQR or NDH-2 are functional in these conditions, a titration with HQNO was performed. Although, the titration curve of the respiratory chain is almost identical to the titration of isolated NQR [24] (Fig 6C), HQNO can also inhibit NDH-2 [65,67,87]. To distinguish between NDH-2 and NQR, the activity was measured with NADH and deamino-NADH. While NQR can use both substrates, NDH-2 is inactive with deamino NADH. The data indicate that the NADH dehydrogenase activity is nearly identical with both substrates, clearly demonstrating that NQR is the main NADH dehydrogenase in *P*. *aeruginosa*. These results are consistent with the BN-PAGE band of 220 kDa having the greatest activity, which contains the six NQR subunits, including the fluorescent subunits B and D. Although the 100 kDa band also has significant NADH dehydrogenase activity, it did not contain NDH-2 and the activity could be due to other flavoproteins present in the sample.

In a recent report, Torres, *et al*. [15] showed that *P*. *aeruginosa* mutants lacking complex I are unable to grow under anaerobic conditions, are less sensitive to gentamycin and showed slightly reduced virulence compared to the wild-type strain, which might indicate that this respiratory complex plays a major role. This conclusion contrasts sharply with the lack of effect of rotenone in our experiments. Interestingly, the authors also showed that individual mutants of complex I, NDH-2 and NQR had no effects on the growth kinetics in several types of growth media, including LB. These results were confirmed by our group, using selected *P*. *aeruginosa* P14 mutant library strains (kindly provided by Dr. Martin Schuster, Oregon State University [88]). As indicated above, mutagenesis studies report the tolerance of microorganisms to the lack of specific enzymes. In the case of *P*. *aeruginosa*, it is not surprising that the other two NADH dehydrogenases can compensate for the lack of any individual enzyme, especially since the cells can grow aerobically even when all three dehydrogenases are removed [15]. Our results show unambiguously that NQR is the preferred NADH dehydrogenase for *P*. *aeruginosa* in the tested conditions, but as shown by the mutagenesis analysis, it is not an essential enzyme.

These results and a recent report by our group [24] suggest that the respiratory chain of *P*. *aeruginosa* has evolved with the ability to resist auto-poisoning. We previously demonstrated that *P*. *aeruginosa* NQR is resistant to HQNO, and that the resistance is conferred by residues Leu151 and Phe155 located in subunit D of the ubiquinone binding site [24]. This characteristic could be an advantage to avoid autopoisoning, since this microorganism actively produces HQNO to eliminate competing bacteria [66,89,90]. Moreover, in a previous report we proposed that NQR could be important for pathogenic bacteria when iron is limiting [91], which is relevant for the infection since the immune system produces iron chelators that deprive pathogens of this essential nutrient. NQR is the ion-pumping NADH dehydrogenase that requires the least amount of iron for its assembly (1 or 2 atoms per molecule [20,21,92,93]), which could allow an active bioenergetic metabolism and cell survival during infection, in particular when the immune response has been mounted.

The main conclusions of this paper are summarized in Fig 1, showing that NQR is the main NADH dehydrogenase in LB (Fig 1B) and mAUM (Fig 1C), that succinate dehydrogenase also plays a major role and that the main terminal oxidases used by *P*. *aeruginosa* are CYO and COx in LB and mAUM media, respectively. Our data offers a clear understanding of the organization of the respiratory chain in physiologically relevant conditions and suggests that the apparent redundancy in the number and type of dehydrogenases and oxidases could be an advantage that allows the cells to avoid autopoisoning by HQNO and cyanide, which *P*. *aeruginosa* actively produces to inhibit the respiratory enzymes of the human host and of competitor bacteria [51,52,97,61,66,67,89,90,94–96, 97].

## Supporting information

S1 Table Proteins identified in the 30 and 46 kDa of the 2D- PAGE gel of LB and mAUM membranes. The 30 kDa fluorescent band was obtained after running a second dimension (2D SDS PAGE) of the 200 kDa band obtained after BN PAGE that contained NADH dehydrogenase activity. The 46 kDa band was obtained after running a second dimension (2D SDS PAGE) of the 100 kDa band obtained after BN PAGE that contained NADH dehydrogenase activity.
(DOCX)

S1 Fig (TIF)
