# Supplementary material for: The aerobic respiratory chain of Pseudomonas aeruginosa cultured in artificial urine media: Role of NQR and terminal oxidases
Source: PLoS One. 2020 Apr 23;15(4):e0231965. doi: 10.1371/journal.pone.0231965 (PMC7179901; doi:10.1371/journal.pone.0231965)
Supplement: S1 Table — The 30 kDa fluorescent band was obtained after running a second dimension (2D SDS PAGE) of the 200 kDa band obtained after BN PAGE that contained NADH dehydrogenase activity. The 46 kDa band was obtained after running a second dimension (2D SDS PAGE) of the 100 kDa band obtained after BN PAGE that contained NADH dehydrogenase activity. (DOCX) [file pone.0231965.s001.docx]

**Supplementary Table I.** Proteins identified in the 30 and 46 kDa of the 2D- PAGE gel of LB and mAUM membranes. The 30 kDa fluorescent band was obtained after running a second dimension (2D SDS PAGE) of the 200 kDa band obtained after BN PAGE that contained NADH dehydrogenase activity. The 46 kDa band was obtained after running a second dimension (2D SDS PAGE) of the 100 kDa band obtained after BN PAGE that contained NADH dehydrogenase activity.

| Identified Proteins | Accession Number | Alternate ID | Molecular Weight |
| --- | --- | --- | --- |
| **30 kDa 2D SDS fluorescent band (200 kDa BN Page)** |  |  |  |
| Ubiquinone oxidoreductase, Na^+^-translocating, B subunit | NQRC_PSEAE | nqrC | 44 kDa |
| Ubiquinone oxidoreductase, Na^+^-translocating, C subunit | NQRB_PSEAE | nqrB | 28 kDa |
|  |  |  |  |
| **46 kDa 2D SDS PAGE (100 kDa BN PAGE)** |  |  |  |
| Outer membrane protein assembly factor BamB | BAMB_PSEAE | bamB | 40 kDa |
| 50S ribosomal protein L6 | RL6_PSEA7 | rplF | 19 kDa |
| Esterase EstA | ESTA_PSEAE | estA | 70 kDa |
| Elongation factor Tu | EFTU_PSEA7 | tuf1 | 43 kDa |
| Porin D | PORD_PSEAE | oprD | 48 kDa |
| Multidrug resistance protein MexA | MEXA_PSEAE | mexA | 41 kDa |
