# Supplementary figures and images for: The aerobic respiratory chain of Pseudomonas aeruginosa cultured in artificial urine media: Role of NQR and terminal oxidases
Source: PLoS One. 2020 Apr 23;15(4):e0231965. doi: 10.1371/journal.pone.0231965 (PMC7179901; doi:10.1371/journal.pone.0231965)

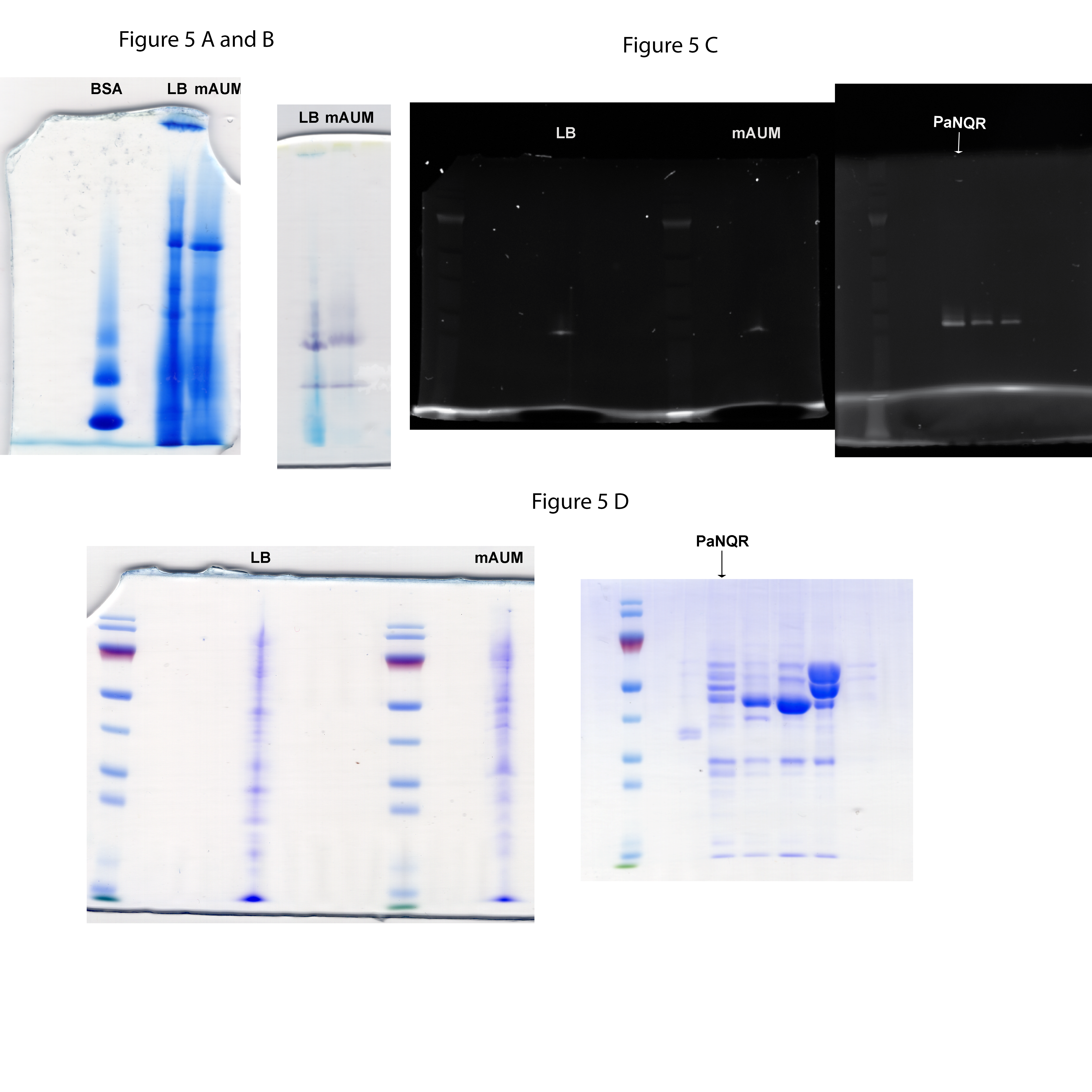

Supplement: S1 Fig — (TIF) [file pone.0231965.s002.tif]
